# Telemedicine use by neurosurgeons due to the COVID-19 related lockdown

**DOI:** 10.1016/j.bas.2021.100851

**Published:** 2021-12-04

**Authors:** Pravesh S. Gadjradj, Roshni H.S. Matawlie, Biswadjiet S. Harhangi

**Affiliations:** a Department of Neurological Surgery, Weill Cornell Brain and Spine Center, New York, NY, USA; b Department of Neurosurgery, Erasmus MC: University Medical Center Rotterdam, the Netherlands

**Keywords:** Covid-19, Telemedicine, Neurosurgery

## Abstract

**Introduction:**

Due to COVID-19 related restriction, the use of telemedicine has increased tremendously. With this increase, an evaluation in the neurosurgical field seems appropriate.

**Research question:**

To what extent has telemedicine made its way in neurosurgical practice during the COVID-19 pandemic?

**Material and methods:**

A 29-question survey was distributed among members of the congress of neurological surgeons regarding the respondents demographics the current level of COVID-19 restrictions, the current use of telemedicine and potential difficulties and consequences of telemedicine for patient care.

**Results:**

The average number of weekly outpatient visits decreased with 31 visits to a mean of 15 visits per week, while the average number of surgeries performed decreased with 5 to a mean of 2 procedures per week. On average 60% of the normal consultations have been converted to telehealth consults. Telemedicine was expected to increase the ability to quickly meet patients for urgent appointments (70%) but was also expected to decrease the quality of the relationship (56%) between practitioners and patients. The biggest difficulties due to use of telemedicine were the inability to perform physical examination (42%) followed by the inability of patients to use technology (24%) and working with elderly patients (20%).

**Discussion and conclusion:**

Telemedicine, however, comes with concerns regarding the quality of the relationship between patients and practitioners and regarding accessibility among certain patient groups. With these concerns, areas of improvement and further research are indicated. Due to the COVID-19 pandemic, telemedicine has become an integral part of the neurosurgical healthcare.

## Highlights

• Due to COVID-19 related restriction, the use of telemedicine among medical specialties has increased tremendously. With this increase, an evaluation in the neurosurgical field seems appropriate.
• On average 60% of the normal consultations have been converted to telehealth consults. Telemedicine was expected to increase the ability to quickly meet patients for urgent appointments (70%) but was also expected to decrease the quality of the relationship (56%) between practitioners and patients.
• The biggest difficulties due to use of telemedicine were the inability to perform physical examination (42%) followed by the inability of patients to use technology (24%) and working with elderly patients (20%).
• Due to the COVID-19 pandemic, telemedicine has become an integral part of the neurosurgical healthcare.

## 1 Introduction

Within a few weeks the corona virus disease 2019 (COVID-19) pandemic demanded a quick adaptation of many medical specialties (Barsom et al., 2020). To maintain sufficient physical distance and to downscale on resources, change was not only visible in the different approach that was needed to provide health care services, but also in the way education was continued during the pandemic (Barsom et al., 2020; Zu et al., 2020; Greven et al., 2020; LoPresti et al., 2020; Deora et al., 2020). Telemedicine is a way to provide health care services at a distant, using various ways of technology for exchanging valid information that is medically related (Organization, 2010). Apart from being a necessity, this change can also be an opportunity to evaluate the usage of telemedicine in the medical field of neurosurgery. The use of telemedicine itself, however, is not a new concept in Neurosurgery. For instance, previous research on the applicability of telemedicine Neurosurgery has shown that telemedicine leads to faster diagnosis of stroke, which may lead to improved patient outcomes due to increased applicability of tissue plasminogen activator (Mouchtouris et al., 2020; Chalouhi et al., 2013; Zanaty et al., 2014; Meyer et al., 2008).

Even though due to COVID-19 the shift towards the use of telemedicine was a necessary step, the use of telemedicine in general has many advantages for patients and practitioners. For instance due to decreased costs by saving time and nullifying mileage (Hayward et al., 2019), reducing waiting time for referrals (Caffery et al., 2016) and even higher patient satisfaction and better patient care (Planchard et al., 2020; Dellifraine and Dansky, 2008). Other observations were a positive effect on decision-making (Hayward et al., 2019), increased accessibility and participation to meeting, conferences, multidisciplinary patient discussions and educational sessions (Deora et al., 2020; Meyer et al., 2008; Planchard et al., 2020). Telemedicine also created new initiatives such as student telehealth hotspotting to provide wellness calls for patients with e.g., higher risk of social isolation (Kaplan, 2021; Finkelstein et al., 2020).

Aside from these advantages, telemedicine may also pose some disadvantages such as difficulties to perform physical examination (Kaplan, 2021; Powell et al., 2017). Other disadvantages may be difficulties to establish an emotional connection with patients on distance or disparity in access to medical care due to the requirement of a computer and broadband internet connection to use telemedicine (Kaplan, 2021; Bergman et al., 2020; Perzynski et al., 2017). Furthermore, the use of internet and computers may also be difficult for older patients which may also negatively influence their access to telemedicine (Levy et al., 2015).

The advantages together with the increased implementation of telemedicine due to COVID-19, may lead to a more permanent place of telemedicine in the medical world even after the pandemic. These prospects emphasize the need to assess and optimize the use of telemedicine to further implement the technology responsibly (Planchard et al., 2020). Preliminary data suggest that geographical variation in the use of telemedicine and some shortcomings of telemedicine among neurosurgeons worldwide (Gadjradj et al., 2020). By the means of this full report of an international survey, we aim to give an overview of the use of telemedicine due to COVID-19, the difficulties encountered with the use of telemedicine and the consequences of the use of telemedicine for patient care.

## 2 Methods

Based on the literature a 29-question survey was created (Zu et al., 2020; Nair et al., 2020; Ray et al., 2017). The survey consisted of four parts (Barsom et al., 2020): demographics of the respondents (Zu et al., 2020); the current level of COVID-19 related restrictions (Greven et al., 2020); the use of telemedicine and (LoPresti et al., 2020) the (potential) difficulties and consequences of telemedicine for patient care. The demographics-section contained questions about the function and specialty, years of clinical experiences, country of employment, age, gender, the average number of outpatient visits per week and the average number of surgical procedures per week before the start of the pandemic. The second part contained questions on the average number of outpatient visits and surgical procedures per week during the COVID-19 pandemic. Additional questions were asked on the level of COVID-19-related restrictions at their institution, and the level of involvement in regular and COVID-19 related patient care. The third section contained questions regarding remote access to medical records, laboratory and radiology data and if patients are currently council using telemedicine. The fourth path contained three questions on how respondents thought the increased use of telemedicine affects patients, themselves and their practice. Furthermore, respondents were asked to comment on the difficulties encountered with telemedicine and to estimate the influence of telemedicine on different aspects of the quality of the care given.

The survey was distributed among the members of the Congress of Neurological Surgeons using SuveryMonkey (Palo Alto, California, USA). The survey was distributed on May 3, 2020, followed by two-weekly reminders till July 2020. The Statistical Package for Social Sciences, version 21.0 for Windows (SPSS, Inc.) was used to analyze the generated data of the survey. The data descriptive statistics were used to present the available data and to display the available data, valid percentages were used. Statistical significance was set at 0.05.

## 3 Results

### 3.1 Respondents

**Fig. 1:**
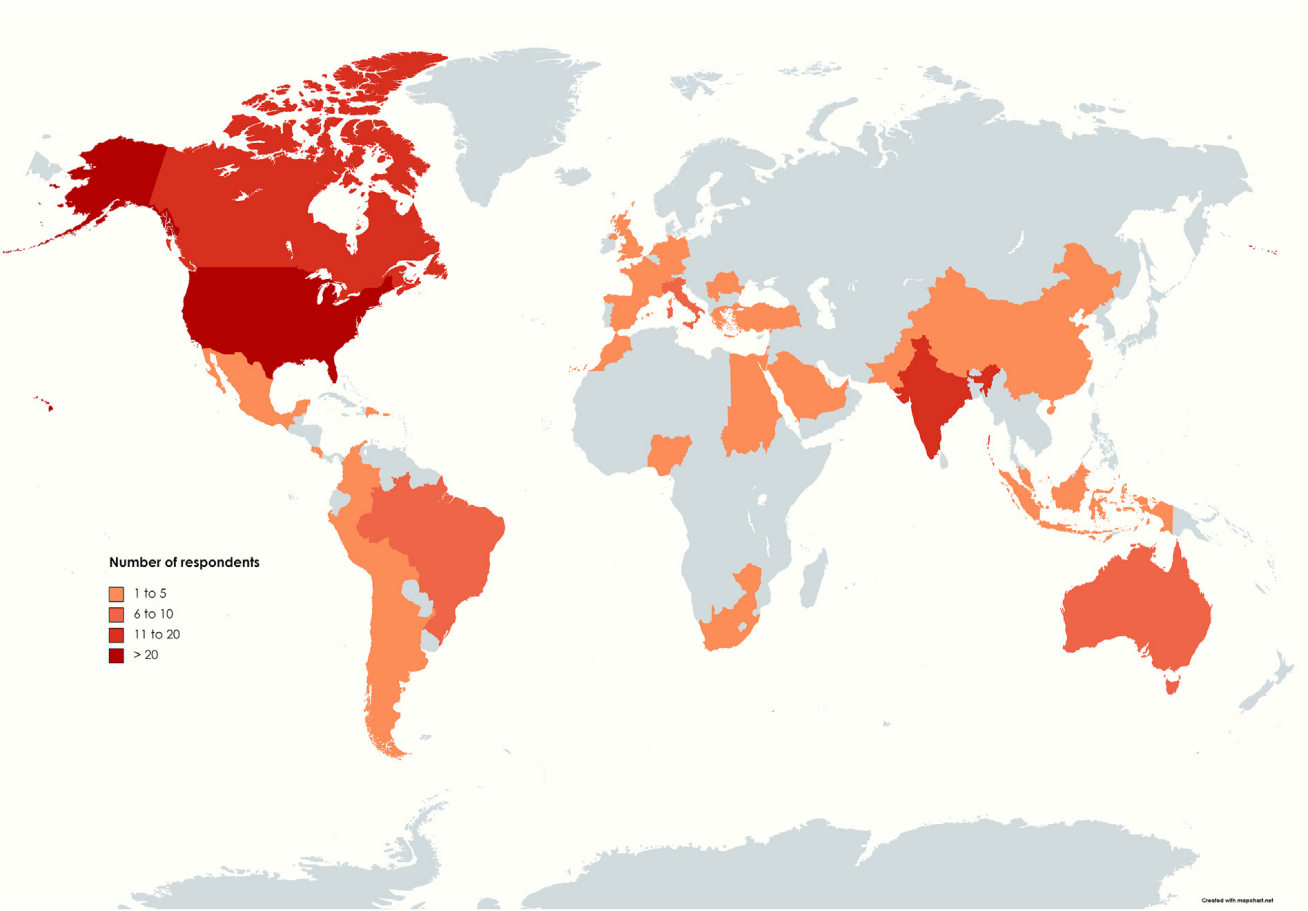
Geographical distribution of the survey respondents.

A total of 363 out of the 5625 approached members replied leading to a response rate of 6.5%. Respondents were employed in a total of forty-three countries with most of the respondents being based in the U.S.A. (see Fig. 1). Most respondents were either neurosurgeon (94.2%) or neurosurgical resident (5.2%), while 11.6% of the respondents overall were female (see Table 1). Before the COVID-19 crisis, respondents had an average of 44.5 ​± ​53 outpatient visits per week, while performing an average of 6.8 ​± ​5.1 surgical procedures weekly.

**Table 1:** Demographics of respondents.

	N (%)		N (%)
**Function**	363	**Gender**	361
Neurosurgeon	342 (94.2%)	Male	319 (88.4%)
Neurosurgeon in training	19 (5.2%)	Female	42 (11.6%)
Other	2 (0.6%)		
		**Specialty**	
**Age**	361	Epilepsy	30 (8.3%)
20–29	3 (0.8%)	Functional	39 (10.7%)
30–39	53 (14.7%)	Peripheral nerve	33 (9.1%)
40–49	82 (22.7%)	Pediatric Neurosurgery	67 (18.5%)
50–59	113 (31.3%)	Neuro-oncology	145 (39.9%)
>60	110 (30.5%)	Neurovascular	98 (27.0%)
		Neuro-trauma	120 (33.1%)
**Continent employed**	363	Spine	213 (58.7%)
North America	262 (72.2%)	Skull Base	80 (22.0%)
South America	28 (7.7%)	Other	21 (5.8%)
Europe	19 (5.2%)		
Africa	7 (1.9%)	**Average number of outpatients visits per week (before COVID-19 crisis)**	44.5 ​± ​53.0
Asia and Oceania	47 (12.9%)		

**Years of clinical experience**	363	**Average number of surgical procedures per week (before COVID-19 crisis)**	6.8 ​± ​5.1
1–5	18 (5.0%)		
6–10	40 (11.0%)		
11–20	91 (25.1%)		
>20	214 (59.0%)		

### 3.2 Current situation

During the COVID-19 crisis, the average number of outpatient visits per week decreased to 14.7 ​± ​14.4 (mean difference −30.7, 95% CI −36.2 to −25.2) while the average number of surgeries performed decreased to 2.3 ​± ​2.3 (mean difference −4.6, 95% CI −5.1 to −4.1) per week (see Table 2). For most respondents (59.7%), all non-essential visits and procedures needed to be postponed while 9.0% of the respondents had experienced no COVID-related restrictions. Furthermore, 21.7% of the respondents were requested to perform guard duties on the ICU, ER or Internal Medicine ward. Overall, 85.1% was still able to physically see patients or to perform surgery. Of current patient group consultations, the majority of 47.7% consisted of emergencies and 40.2% consisted of suspected high-grade neuro-oncology. Overall, only 2.4% of the respondents was not performing surgery at all. Of the neurosurgeons that did perform surgery, 33.6% performed surgery for neuro-oncology, 24.1% performed only trauma surgery and 23.8% for degenerative spinal surgery.

**Table 2:** Responses regarding the current situation.

	N (%)		N (%)
Average number of outpatients visits per week (during COVID-19 crisis)	14.7 ​± ​14.4	Current patient group consultations	
		Trauma	137 (37.7%)
		Infection	113 (31.1%)
**Average number of surgical procedures per week (during COVID-19 crisis)**	2.3 ​± ​2.3	Suspected high-grade neuro-oncology	146 (40.2%)
		Emergencies	172 (47.4%)
		All patients who come	153 (42.1%)
**Level of COVID19-related restrictions**	355		
All non-essential visits and procedures need to be postponed	212 (59.7%)	**Currently performed surgeries**	
		Only trauma surgery	69 (24.1%)
Only some non-essential visits and procedures need to be postponed	111 (31.3%)	Non-oncological peripheral nerve surgery	1 (0.3%)
		Functional neurosurgery	11 (3.8%)
No restrictions, visit and elective procedures carried out as normal	32 (9.0%)	Hydrocephalus	34 (11.9%)
		Degenerative spinal surgeries	68 (23.8%)
		Neuro-oncological related care	96 (33.6%)
**(Physically) seeing/operating patients**	356	I am not performing any surgeries	7 (2.4%)
Yes	303 (85.1%)		
No	53 (14.9%)	**Requested for guard duties in Internal Medicine/ICU/Emergency**	355
		Yes	77 (21.7%)
		No	278 (78.3%)

### 3.3 Use of telemedicine

Overall, 87.6% of the respondents had remote access to patient data and used telemedicine for consultations Table 3). Telemedicine was mostly used for consultations of known and new patients (56.2%) and in lesser extent for only known patients (47.1%), lectures (32.8%), research meetings (27.5%), peer consultations (22.9%) and emergencies (14.9%). Telemedicine was applied by the means of telephonic consults (52.6%), video consultations (58.1%) and Email/WhatsApp/other social media applications (22.6%). For almost all respondents (96.7%) the use of telemedicine has increased during the pandemic. On average, 59.7% of the normal consultations have been converted to telehealth consults. Overall, 76.2% of the patients are receptive to telemedicine, 16.2% is neutral and 5.1% is not receptive.

**Table 3:** Responses on the application of telemedicine.

	N (%)		N (%)
**Has remote access to monitor medical records, laboratory and radiology data**	339	**Specify usage of telemedicine for consultations**^a^	
Yes	297 (87.6%)	Telephonic consults	191 (52.6%)
No	42 (12.4%)	Email/WhatsApp/Telegram/Other social media applications	82 (22.6%)
	
**Usage of telemedicine for consultation**	340	Video consultations (Zoom, Skype,Teams)	211 (58.1%)
Yes	298 (87.6%)	Other	26 (7.2%)
No	42 (11.6%)	None as of now, but I plan to start soon	2 (0.6%)
		I do not plan to do any of the above	0
**Increased usage of telemedicine during COVID-19 crisis**	275		
		**How receptive are patients to telemedicine**	277
Yes	266 (96.7%)		
No	9 (3.2%)	Receptive	211 (76.2%)
		Neutral	45 (16.2%)
**Specification usage telemedicine**^a^		Not receptive	14 (5.1%)
Consultation for known patients	171 (47.1%)	Other	7 (2.5%)
Consultations for known patients and new patients	204 (56.2%)		
		**Opinions about the risks faced with regards to COVID19**	320
Emergency	54 (14.9%)		
Lectures	119 (32.8%)	Equal risk of contracting COVID19 compared to other specialties when it comes to examining patients	160 (50.0%)
Peer consultation	83 (22.9%)		
Research meetings	100 (27.5%)		
		Higher risk of contracting COVID19 compared to other specialties when it comes to examining patients	37 (11.6%)
**Are you comfortable to consult and plan surgery based on telemedicine visits**	278		
Yes	147 (52.9%)	Lower risk of contracting COVID19 compared to other specialties when it comes to examining patients	123 (38.4%)
No	105 (37.8%)		
Other	26 (9.4%)		

**Are there restrictions as to where you are allowed to provide telemedicine services**	277		
	
Yes, it should be from the hospital	63 (22.7%)		
No, it's allowed to work from home	214 (77.3%)		

**Percentage of normal consultations converted to telehealth consults**	278		
	
0–25%	66 (23.7%)		
26–50%	57 (20.5%)		
51–75%	39 (14.0%)		
76–100%	116 (41.7%)		

a Multiple answers possible.

### 3.4 Difficulties experienced using telemedicine and consequences of telemedicine on patient care

**Fig. 2:**
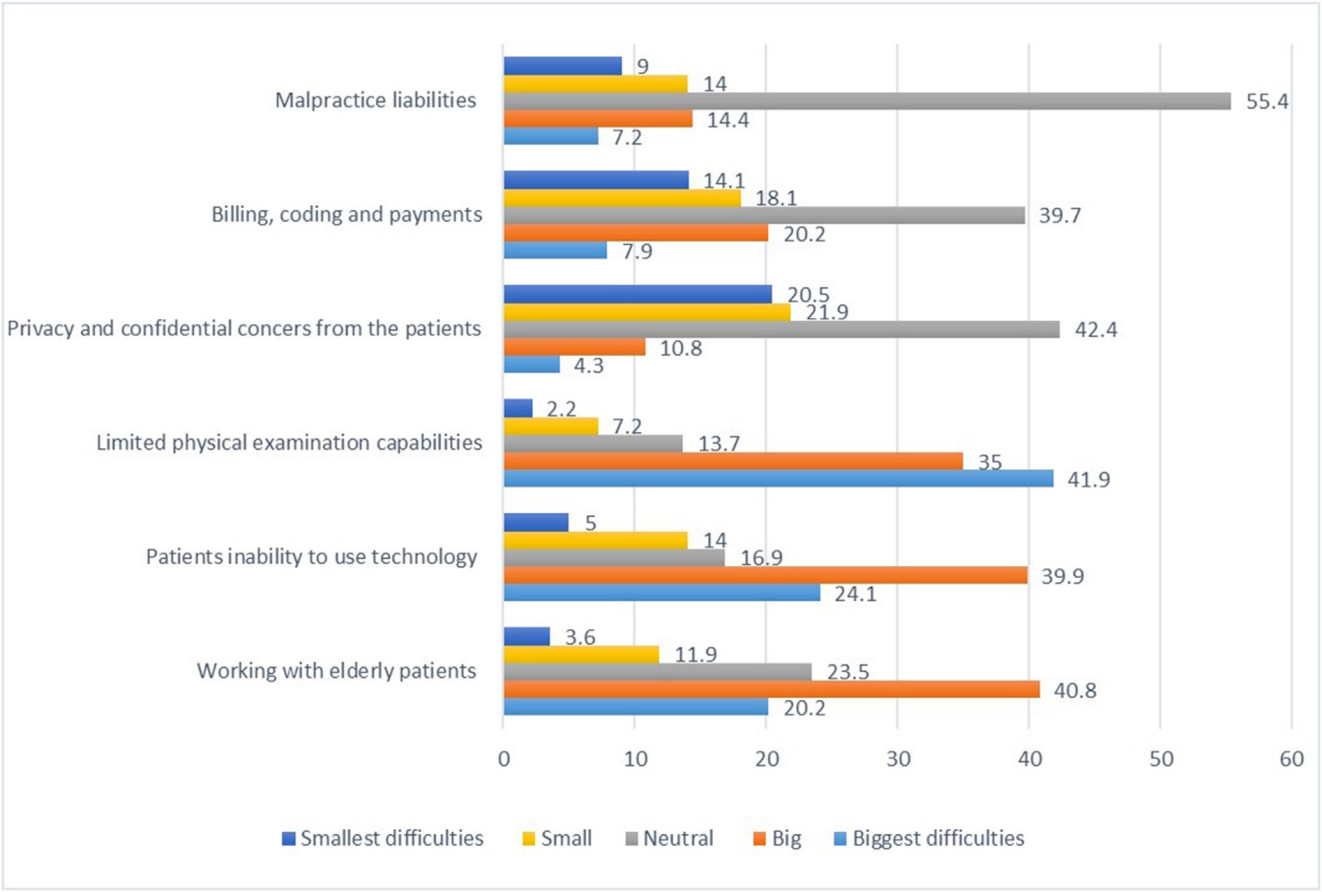
Difficulties encountered during the practice of telemedicine.

Fig. 2 gives an overview of the difficulties neurosurgeons encounter during the usage of telemedicine. The biggest difficulty experienced with telemedicine is the limited capability to perform physical examination (by 41.9%). This was followed by the inability of patients to use technology (24.1%) and working with elderly patients (20.2%). Smaller difficulties were experienced with privacy concerns and financial aspects, while most neurosurgeons (55.4%) were neutral regarding difficulties due to malpractice liabilities.

**Fig. 3:**
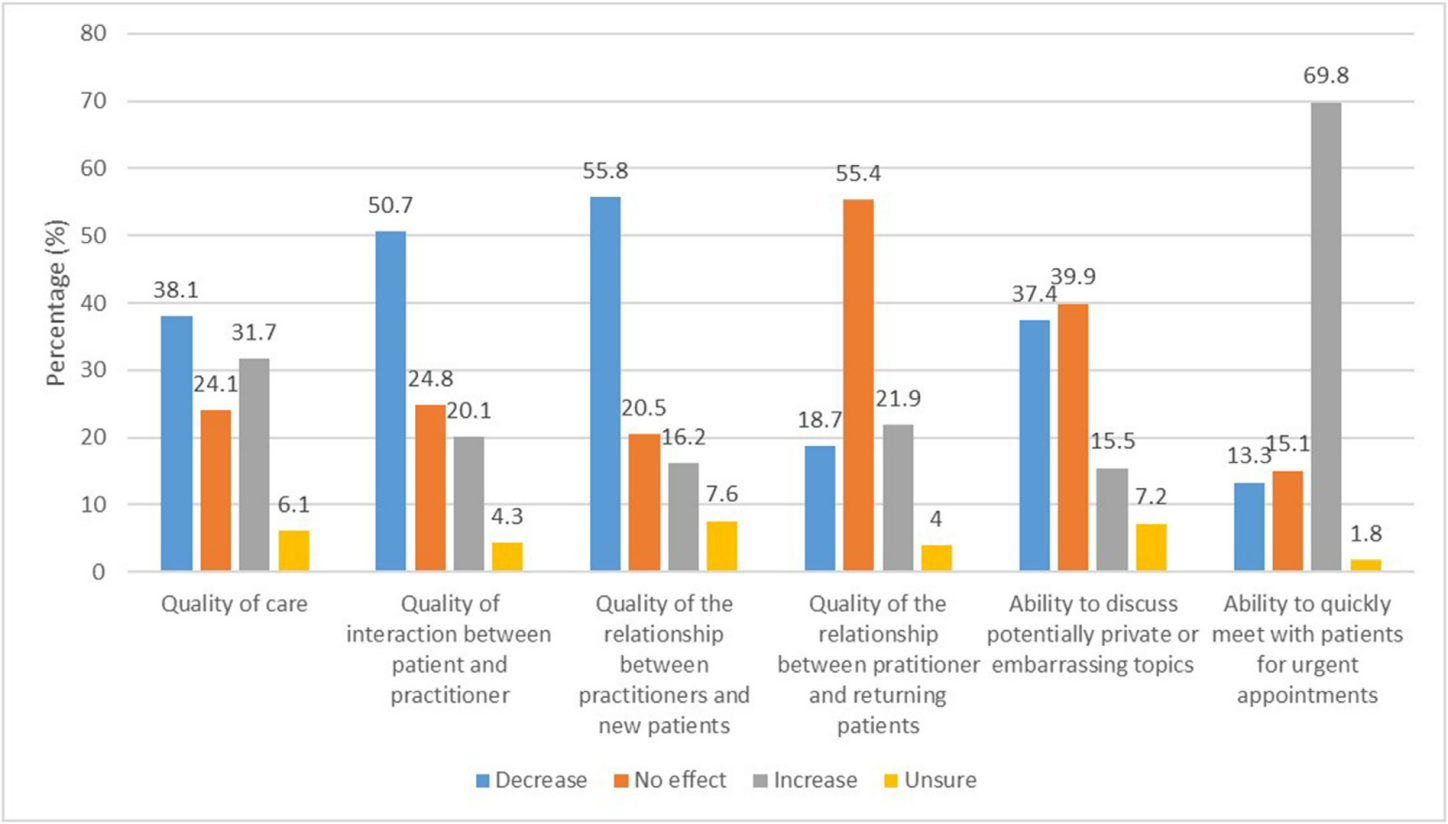
Respondents' opinion on the consequences of telemedicine application on different aspects of patient care.

Respondents’ opinion on the consequences of telemedicine application on different aspects of patient care are depicted in Fig. 3. Overall, the majority (69.8%) expected an increase in the ability to quickly meet patients for urgent appointments. However, majorities also expected declines in the quality of the relationship between practitioners and new patients (55.8%) and the quality of interaction between practitioners and patients (50.7%). For returning patients, the majority (55.4%) believed that the use of telemedicine would not influence the quality of the relationship. Overall, respondents were more divided on the effect of telemedicine on the quality of care in general. Table 4 gives an overview of a selection of open answers on how the increased use of telemedicine has affected patients and surgeons themselves together with their practice.

**Table 4:** Overview of selected open answers on questions regarding how the increased usage of telemedicine has affected (Barsom et al., 2020) patients and (Zu et al., 2020) themselves and their practice.

**How do you think these changes affect your patients?**
*“Decreased accuracy of detailed physical exams, but improved access for many patients that travel long distances.”*
*“This is a positive change. My patients are no longer required to come to the office and I am able to see new consultations by video consultation. This has decreased the amount of travel my patients are responsible for.”*
*“Delays in management and delays in presentation to the hospital for some emergencies.”*
*“Easier access for patients. Difficult to show imaging studies to patients. Not able to perform a neurological exam.”*
*“For patients traveling a great distance or who are in medically poor areas of the US, allows better and more timely access and evaluation by neurosurgery.”*
*“I think it's stressful. Some patients have shown up for their telemedicine visit while driving a car with the child not in the car and it was the child's visit. Somethings are very hard to see and examine, particularly in infants. Well somethings lend themselves well to telemedicine, I remain extremely concerned that other things do not, and that people are too scared to come in, and that we are not able to do a good job and evaluating them over a camera.”*

**How do you think these changes affect you and your practice?**
*“This has had a positive impact. Based on insurance restrictions I cannot order imaging without seeing a patient which results in 2 trips to the office. Now, I am able to see the patient in consultation over video visit and then order the imaging. Then the patient is able to come to clinic the same day as imaging is done or go home and follow up over video visit.”*
*“Mixed: lack of graded motor and reflex exam is limiting, but, by same token, being able to still provide care to people despite strict precautions is good.”*
*“I will continue telemedicine for post ops, follow ups (image review), and patients who live farther away. I will use this as a screening appointment to assess need for in-office exam and need for further imaging/management before offering surgery.”*
*“Increased time required to coordinate outpatient communication, improved efficiency and access to patients for clinic.”*
*“It is extremely time consuming. It takes twice as long to do a telemedicine visit as it does an in person visit, and then there is the backtracking to check on x-rays and labs which need to be performed at other places, and then need to be called back and discussed. Patients have much more limited access to viewing their images unless I hold my computer up to another computers' camera.”*

## 4 Discussion

The current study presents the results from a survey among 363 members of the CNS regarding the use of telemedicine, the difficulties experienced with the use of telemedicine and the consequences of telemedicine application on patient care.

At the time of answering the survey, the average number of weekly outpatient visits decreased with 31 visits to a mean of 15 visits per week, while the average number of surgeries performed decreased with 5 to a mean of 2 procedures per week. Overall, 85% of the respondents were still able to physically see patients or to perform surgery. On average 60% of the normal consultations have been converted to telehealth consults. Most used forms of telemedicine were video consultations and consultations by telephone. From the perspective of the neurosurgeon, 76% of the patients were receptive to telemedicine.

Telemedicine was expected to increase the ability to quickly meet patients for urgent appointments but was also expected to decrease the quality of the relationship (56%) between practitioners and patients. The biggest difficulties faced with the use of telemedicine were the limited capability to perform physical examination, followed by the inability of patients to use technology and working with elderly patients.

### 4.1 Comparison with other studies

As the use of telemedicine has increased massively during the COVID-19 pandemic, so has the research on the use of telemedicine (Tandon et al., 2021; Shafi et al., 2021; Swiatek et al., 2021; Ryu et al., 2021; Zengin et al., 2021; Lovecchio et al., 2020; Mohanty et al., 2020; Riew et al., 2021)*.* Table 5 gives an overview of eight published survey results on the use of telemedicine during the COVID-19 pandemic among neurological and spine surgeons. Most of these studies were conducted during summer 2020 among mostly members of professional organizations. In general, these studies conclude that surgeons are receptive of telemedicine to perform consultations but that some concerns remain such as patients’ preferences for in-person visits, the limited capabilities to perform physical examination and technical issues. These findings are in line with our current study.

**Table 5:** Schematic overview of neurosurgical surveys regarding telemedicine use during the COVID-19 Pandemic.

Author, year	Aim	Questions	Period conducted	Study population	Distribution via	Response rate	(partial) Conclusion
Mohanty 2020	*“To investigate both patient and provider satisfaction with telemedicine and its strengths and limitations in outpatient neurosurgery visits.”*	11	March–July 2020	Neurosurgical providers practicing telemedicine at the authors center or a similar academic tertiary center in the country.	E-mail	47.1% (40/85)	*“Although the authors' transition to telehealth was both rapid and unexpected, most providers and patients reported positive experiences with their telemedicine visits and found telemedicine to be an effective form of ambulatory neurosurgical care. Not all patients preferred telemedicine visits over in-person visits, but the high satisfaction with telemedicine by both providers and patients is promising to the future expansion of telehealth in ambulatory neurosurgery.”*
Lovecchio 2020	*“To utilize data from a global spine surgeon survey to elucidate* (Barsom et al., 2020) *overall confidence in the telemedicine evaluation and* (Zu et al., 2020) *determinants of provider confidence.”*	42	May 2020	Members of AOSpine	E-mail	485 surgeons	*“Spine surgeons are confident in the ability of telemedicine to communicate with patients, but are concerned about its capacity to accurately make physical exam-based diagnoses. Future research should concentrate on standardizing the remote examination and the development of appropriate use criteria in order to increase provider confidence in telemedicine technology.”*
Deora 2020	*“Therefore, it is imperative to evaluate whether the pandemic has had a discernible effect on health care providers, especially in terms of practice modifications in private establishments and publicly funded hospitals, the emotional impact on the surgeon, and the influence of social media on the psyche of the surgeon.”*	26	May 2020	Neurosurgeons from the Indian subcontinent.	Social media groups, focused e-mail lists and direct messaging platforms.	17.6% (176/1000)	*“Although telemedicine has not been as widely adopted as expected, online education has been favorably received.”*
Riew 2021	*“To explore international perspectives of spine providers on the challenges and benefits of telemedicine.”*	42	May 2020	Members of AOSpine	E-mail	485 surgeons	*“Spine surgeons are supportive of the benefits of telemedicine, and only a small minority experienced technical issues. The decreased ability to perform the physical examination was the top challenge and remains a major obstacle to virtual care for spine surgeons around the world, although interestingly, 61.4% of providers did not acknowledge this to be a major challenge.”*
Ryu 2021	*“This study aimed to characterize user experiences of neurosurgeons and advanced practice providers focusing on perceived utility and barriers of telemedicine in management of elective neurosurgical patients during COVID-19.”*	14	COVID-19 period	Health care providers of neurosurgical care in a single center of the U.S.	Online survey	82.4% (14/17)∗	*“During the COVID-19 period, telemedicine was heavily relied on to ensure the continuation of perioperative care for patients with elective neurosurgical pathologies. While clinicians identified numerous barriers for current telemedicine platforms, the use of telemedicine will likely continue, as it has provided unique benefits for patients, clinicians, and hospitals.”*
Swiatek 2021	*“To assess spine surgeon reliance on virtual medicine during the pandemic and to discuss the future of virtual medicine in spine surgery.”*	73	March–April 2020	Members of AOSpine	E-mail	23.7% (902/3805)	*“COVID-*19 has *changed spine surgery by triggering rapid adoption of virtual medicine practices. The demonstrated global interest in virtual medicine suggests that it may become part of the “new normal” for surgeons in the postpandemic era.”*
Shafi 2021	*“To utilize a global survey to elucidate spine surgeons' perspectives towards research and resident education within telemedicine.”*	42	May 2020	Members of AOSpine	E-mail and personal reach-out	485 surgeons	*“Our study of spine surgeons worldwide noted high agreement among specialists for the implantation of telemedicine in trainee curricula, underscoring the global acceptance of this medium for patient management going forward.”*
Tandon 2021	*“To explore the geographical (continent-based) differences in telemedicine practices to learn about the problems faced in different regions”*	30	August 2020	Neurosurgeons practicing across the world.	E-mail and social media platforms	23.8% (286/1200)	*“Telemedicine in neurosurgery is a viable alternative to physical outpatient services during the COVID-19 pandemic and could potentially play a vital role after the pandemic.”*

### 4.2 Improving acceptance and quality of telemedicine

In the literature multiple recommendations are published on how to improve quality and how to improve the acceptance of telemedicine among both patients and health care providers (Zu et al., 2020; Hsueh et al., 2021; Gachabayov et al., 2021; Lambert et al., 2021). In a well-written review paper by LoPresti et all, seven common problems with solutions were discussed on how to effectively apply telemedicine. These problems involve:

1. Patient access: elder patients or patients with language barriers may find more difficulties in using telemedicine or effectively communicating symptoms. This can be solved by having a helpdesk or by using the telephone while treating patients that are not able to set-up the proper telemedicine necessities.
2. Provider access: this can be solved by having the correct equipment, rooms and software available to providers.
3. Limitations in performing physical/neurological examination: although it is not feasible to perform all parts of the physical examination through telemedicine, initiatives have been taken to implement standardized remote physical examinations (Iyer et al., 2020).
4. Privacy/confidentiality concerns: providing patients information on patient confidentiality is key in this.
5. Potential issues with billing: billing issues may be very dependent on the health care system of the provider, but since the start of the pandemic many issues regarding billing of telemedicine services may have been solved.
6. Malpractice liabilities: health care providers should make sure that if they cannot make adequate decisions in managing patients through telemedicine, that in-person visits should be considered.
7. Restrictions due to medical licensure: this may be very dependent on the location of the health care provider.

### 4.3 Patient satisfaction

In the current study, most of the patients are receptive to telemedicine from the perspective of the neurosurgeons. However, neurosurgeons also expected that the application of telemedicine had a negative effect on the quality of interaction between patient and practitioner and a negative effect on the quality of the relationship between practitioners and new patients. In this study the patients’ perspective was not evaluated. This belonged, however, to the scope of other studies (Porche et al., 2021; Yoon et al., 2021; Maurer et al., 2021). In their retrospective analysis of survey data of 97 patients that were consulted by telemedicine and 589 patients who had in-person consultations, Porche et al. showed that overall patient satisfaction did not differ between both patient groups (36). Satisfaction scores on other domains such as accessibility to care and contact with the care provider also did not differ between both patient groups. In a survey among 176 patients at a spine clinic, Maurer et al. showed that patients that who had to travel further distances to the hospital may be associated with favoring telehealth and that most spine patients preferred in-person appointments over virtual appointments^34^. However, in a larger prospective analysis of 310 patients that underwent a telemedicine visit in a U.S. based Neurosurgery department, Yoon et al. measured satisfaction with telemedicine with questionnaires distributed after their consultation (Yoon et al., 2021). On a scale of ranging from 1 to 7 (= very satisfied), the average score was 6.3. Of all the telemedicine consultations, only 1 patient (0.3%) was sent to the emergency department and 94 patients (30.3%) had imaging ordered, indicating that most of the patients could be successfully consulted by telemedicine only. Additional analyses of satisfaction scores among patients who lived at more than 15 miles distance to the hospital versus those who don't, and between returning and new patients, revealed no differences. The prior contrasting with the expectation of neurosurgeons in our current survey.

### 4.4 Limitations

Some limitations must be acknowledged such as the response rate. Even though our response may be somewhat comparable to other surveys conducted among large physician databases such as AOSpine International, the response rate can be rated to be low. A low response rate may induce selection bias, but as the survey did not cover controversial topics, it is not to be expected that specific group of surgeons will be more motivated to reply. As Table 5 shows, quite a few surveys were conducted during the summer 2020 and this might have negatively impacted the response rate. Nevertheless, the absolute number of respondents is high enough to give an impression on the application of telemedicine during the COVID-19 pandemic. Furthermore, the survey response rate might not necessarily be related to the quality of the study (Livingston and Wislar, 2012). Other limitations inherent to the study design, e.g. recall bias, also warrant cautious interpretation.

## 5 Conclusion

Due to the COVID-19 pandemic, telemedicine has become an integral part of the neurosurgical healthcare. Telemedicine, however, comes with concerns regarding the quality of the relationship between patients and practitioners and regarding accessibility among certain patient groups. With these concerns, areas of improvement and further research are indicated.

## Funding

Not applicable.

## Previous presentations

Not applicable.

## Disclosure

All authors (PSG, RHSM and BSH) have no conflicts of interest to disclose.
